# Diagnostic value of effusion adenosine deaminase, γ-interferon release assay and effusion lactatedehy drogenase/effusion adenosine deaminase for tuberculous pleural effusion in patients aged 60 years and above

**DOI:** 10.3389/fcimb.2024.1444238

**Published:** 2024-10-09

**Authors:** Fei Guo, Chen Huimin, Wei Xia, Yilin Xu, Weijiang Jin, Fang Liu

**Affiliations:** ^1^ Department of Laboratory Medicine, The First Affiliated Hospital of Ningbo University, Ningbo, Zhejiang, China; ^2^ Department of Respiratory and Critical Care Medicine, The First Affiliated Hospital of Ningbo University, Ningbo, Zhejiang, China

**Keywords:** tuberculous pleural effusion, age, adenosine deaminase, interferon gamma releasing assays, diagnosis, ratio

## Abstract

**Background:**

China is experiencing rapid growth in its population of older adults, which may lead to increased susceptibility to tuberculous pleural effusion (TPE) due to age-related changes in the immune system. This study aimed to investigate the diagnostic potential of multiple biomarkers in individuals aged 60 years and above with pleural effusion.

**Methods:**

A total of 519 adult patients from Ningbo First Hospital were included in the study, with 7 biomarkers and their ratios in serum and pleural effusion analyzed using logistic regression analysis. Effusion Adenosine Deaminase(ADA), γ-Interferon Release Assay(IGRA), and Effusion lactatedehy drogenase(LDH)/Effusion ADA were identified as valuable parameters for differentiating TPE from non-TPE, particularly in individuals aged 60 years and older.

**Results:**

Effusion ADA, IGRA, and Effusion LDH/Effusion ADA were identified as valuable parameters for the differential diagnosis of TPE from non-TPE, showing good diagnostic performance in individuals aged 60 years and older. The combined diagnosis of these three indexes achieved the highest diagnostic accuracy for TPE in this age group, with an AUC of 0.925, sensitivity of 85.23%, and specificity of 89.57%.

**Conclusions:**

Overall, the study highlights the importance of using multiple indicators for a combined diagnosis to improve diagnostic efficacy in detecting tuberculous pleurisy in older individuals as for young patients.

## Introduction

According to the latest World Health Organization (WHO) report, the Tuberculosis (TB) incidence rate (new cases per 100 000 population per year) rose by 3.6% between 2020 and 2021 (Bagcchi, 2023). Pulmonary tuberculosis is a serious global public health problem, particularly in developing countries. China has the second-highest TB burden globally, with an estimated 842,000 people with TB (59 per 100,000 population per year), with older adults accounting for a quarter of all cases. China notified 159,100 cases (19% of all notified cases) among older adults (C. Antimicrobial Resistance, 2022), and a consistently increasing trend has been observed over the last two decades (Wang and Wang, 2021).

Tuberculous pleural effusion (TPE) is the second most common form of extrapulmonary tuberculosis (Light, 2010; Shaw et al., 2019), and the effusion etiology is complex, particularly in middle-aged and elderly patients (The World Health Organization (WHO) defines elderly people as those aged 60 and above (Mekonnen et al., 2023). Moreover, mass-like lesions or nodules due to TB in the elderly can often be difficult to distinguish from malignant disease or more common bacterial pneumonias (Schaaf et al., 2010). Combining different causative factors, TPE is still the most common cause of exudative effusion in developing countries (Skouras and Kalomenidis, 2016; Jiang et al., 2020). Therefore, with the increase of the aging population, early and accurate diagnosis of TPE is extremely critical for the management of TPE in the elderly.

The definitive diagnosis of TPE relies on detecting *Mycobacterium tuberculosis* in sputum, pleural effusion, or pleural biopsy specimens (Light, 2010), which is difficult because of the low sensitivity of bacterial culture methods (<10%: acid-fast bacilli of pleural fluid, 20–30%: *M. tuberculosis* culture of the fluid). In obscure cases, closed needle pleural biopsy is conducted, however, this invasive method yields only a confirmation of 60–80% for tuberculosis pleurisy (Lee et al., 2014). As a result, researchers have investigated various biomarkers in pleural effusion, such as adenosine deaminase (ADA) and interferon-gamma (IFN-γ) (Porcel, 2018).

In cases of TPE, the host mounts a cellular immune response to pleural lymphocytes, leading to the secretion of ADA2 by monocytes and macrophages, consequently increasing ADA levels (Porcel et al., 2010). This enzyme is commonly utilized in the diagnosis of TPE due to its cost-effectiveness, ease of detection, and widespread availability (Klimiuk and Krenke, 2011; Zhang et al., 2020; Arpinar Yigitbas et al., 2021). However, elevated ADA levels can also be observed in malignant conditions, bacterial pneumonias, and other scenarios, and its diagnostic accuracy may be influenced by age (Abrao et al., 2014; Solari et al., 2017; Korczynski et al., 2019). Therefore, in suspected TPE cases, a combination of ADA measurement and IGRA is preferred as IGRA results are less susceptible to age-related variations.

Lymphocytes from patients with TPE exposed to *Mycobacterium tuberculosis* generate numerous memory lymphocytes in the body. Additionally, upon reexposure to mycobacterial antigens, lymphocytes from individuals infected with tuberculosis release higher levels of IFN-γ, suggesting interferon-gamma as a potential diagnostic marker for tuberculosis infection (Losi et al., 2007). A study reported that IGRA testing was 94.9% sensitive and 96.3% specific detecting tuberculous effusion (Wongtim et al., 1999), potentially outperforming Adenosine Deaminase (ADA) testing in TPE diagnosis (Jiang et al., 2007). Furthermore, the IGRA test of pleural effusion in the diagnosis of TPE may have stable performance in different age groups (Hochberg et al., 2016). Elevated levels of γ-interferon in pleural effusion may hold significant diagnostic value for TPE in older individuals, although the standardized cutoff values remain lacking (Porcel, 2018; Jiang et al., 2020).

With the increasing aging population, there is a growing need to continually enhance the diagnostic methods for TPE in the elderly. In this study, we specifically examined TPE patients aged 60 and above, aiming to investigate the variations in diagnostic performance among different diagnostic indicators and ratios for TPE patients in the older age group.

## Objectives and methods

### Study population

**Figure 1:**
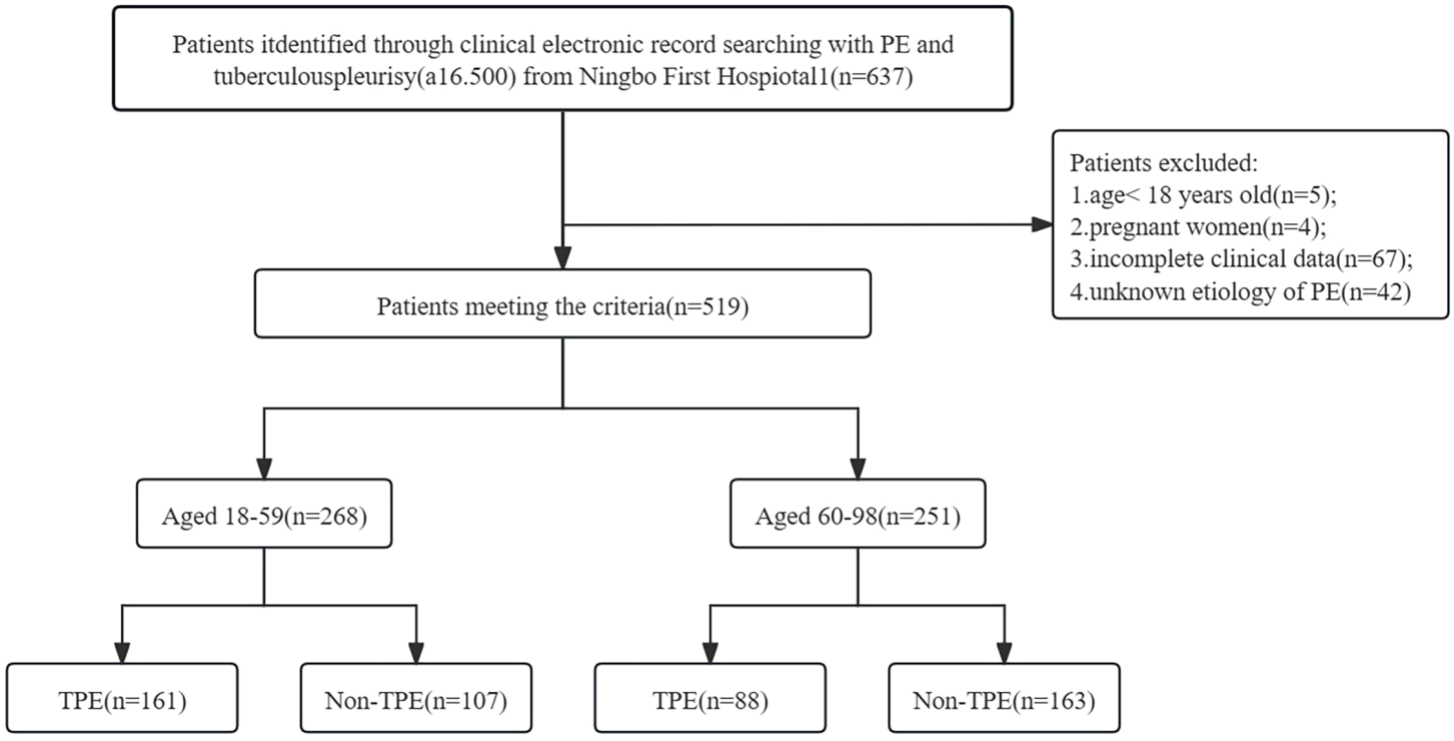
Flow diagram of study selection. PE, Pleural effusion; non-TPE, non-tuberculosis effusion; TB, tuberculosis; a16.500: Disease code from National Clinical Version 2.0.

This study was approved by Ethics Committee of the First Affiliated Hospital of Ningbo University (approval number: 2024087RS). All patients newly diagnosed with pulmonary embolism (PE) between January 2015 and April 2024 were recruited from Ningbo First Hospital. Individuals under the age of 18 and those who declined to provide informed consent were not included in the study. The patient enrollment process is illustrated in **Figure 1**. Included patients were admitted to the hospital for the first time due to pleural effusion. Samples of PE and peripheral blood were collected and analyzed. Data from the initial samples of PE and blood from each patient were examined. Relevant statistics, laboratory results, and clinical characteristics for all patients were extracted from the clinical electronic record system. A total of 519 patients with PE were enrolled, with 249 diagnosed as TPE and 270 as non-TPE. Among these patients, 268 were between 18 and 59 years old, while 251 were aged 60 years and above (refer to **Figure 1**). The following criteria were applied to all participants: (i) Diagnosis of PE was confirmed through ultrasound, chest CT, or X-ray; (ii) All participants underwent cytology, thoracentesis, or pleural biopsy for diagnosis and follow-up (minimum follow-up period of 6 months). Exclusion criteria encompassed: (i) Individuals under 18 years old; (ii) Participants with incomplete clinical data; (iii) Pregnant individuals; (iv) Uncertain clinical diagnosis.

### Standardized diagnostic criteria for TPE and non-TPE

Patients diagnosed and treated for TPE in our hospital were included in our study. The diagnostic criteria for TPE included: (a) Positive culture of effusion or pleural tissue for *Mycobacterium tuberculosis*. (b) Isolation of Mycobacterium tuberculosis from granulomatous inflammation identified in pleural biopsy histology. (c) Presence of granulomatous inflamed tissue in pleural biopsy along with clinical response to antituberculosis therapy (Lin et al., 2009; Bielsa et al., 2013; Botana Rial et al., 2023). A total of 270 non-TPE patients were included in **Table 1**. Thoracoscopic pleural biopsy indicating malignant disease was diagnosed as malignant effusion. No macroscopic empyema was observed in the pleura, and the biochemistry, cell type, and number met the diagnostic criteria for parapneumonic effusion. All patients were diagnosed based on pathology results, clinical examination, and imaging consistent with the respective diseases.

**Table 1:** Diagnosis of non-TPE (N=270).

Diagnosis of Non-TPE	N(%)
Malignant pleural effusion	139(51.48)
Parapneumonic effusion	108(40.00)
Empyema	12(4.44)
Chronic heart failure	8(2.97)
Eosinophilic pleuritis	1(0.37)
Hypergamma globulinemia	1(0.37)
parasitic infection	1(0.37)

### Data capture

All the data of clinical and laboratory, including age, gender, smoking history, and effusion biochemical indexes (Total protein(TP), albumin(ALB), ADA, LDH), peripheral blood indexes (C-reactive protein(CRP), erythrocyte sedimentation(ESR), IGRA), and serum indexes (TP, ALB, ADA, and LDH) (**Table 2**), were retrieved from the clinical electronic record system. This study received approval from the Ningbo First Hospital Ethics Committee, and the requirement for informed consent was waived. We affirm that all methods were conducted in accordance with relevant guidelines and regulations.

**Table 2:** The characteristics of study participants.

Characteristics	Aged 18-59(n=268)	Aged 60-98(n=251)
Diseases[TPE/Non-TPE(TPE%)]	161/107(60.07)	88/163(34.92)
Gender[male/female]	171/98	181/70
Smoke Status	268(40.7)	251(78.2)
Age	44(30-54)	72(67-77)
Serum		
CRP(mg/L)	44.5(16.5-84.8)	32.05(8.75-70.27)
ESR(mm/h)	48(32-65)	42(25-60)
TP(g/L)	67.35(62.73-72.48)	64.65(59.58-70.03)
ALB(g/L)	35.9(32.9-38.82)	33.15(29.98-36.2)
ADA(U/L)	9.8(7.6-13.4)	9.7(7.7-12.6)
LDH(U/L)	183(157-220)	186(154.25-236)
IGRA(pg/mL)	66(3-191)	23(2-119.75)
Effusion		
TP(g/L)	50.5(46.6-54.3)	45.7(38.58-51.1)
ALB(g/L)	28.86(25.82-30.77)	25.55(20.75-28.53)
ADA(U/L)	27.35(12.25-39.25)	12(7.3-28.6)
LDH(U/L)	455(262.5-733.5)	315(204-565)

TP, Total protein; ALB, albumin ADA adenosine deaminase; LDH, lactatedehy drogenase; CRP, C-reactive protein; ESR, erythrocyte sedimentation rate; IGRA, Interferon-γ release assay.

Continuous variables were presented as median and inter quartile rang (IQR, 25th–75th). Categorical variables were presented as number and percentage (n, %).

### PE and blood indexes analysis

The participants in the TB-IGRA experiment used heparin anticoagulant vacuum tubes to collect heparinized anticoagulated whole blood. Culture filter protein 10 (CFP-10) and early secretory antigen 6 (ESAT-6) containing *Mycobacterium tuberculosis* (MTB) specific antigens were added to the test tubes. CFP-10 and ESAT-6 stimulated MTB-specific T lymphocytes to proliferate and release IFN-γ, which was then detected in plasma using enzyme-linked immunoassay (ELISA). The method has a linear range of 2-400 pg/ml, with values of ≤2 being counted as 2. The kit used was provided by Wantai Biopharmaceutical Co., Ltd (Beijing, China). CRP was assayed by Immunoturbidimetry using ARISTO from Guosai Technology Co., Ltd (Shenzhen, China). ESR was assayed with Test1 from the Italian company ALIFAX. PE and serum TP were assayed by the biuret method, ALB by the bromocresol green end point assay method, and LDH by the modified IFCC method using Olympus AU5821 from Beckman Coulter (Suzhou, China). ADA was assayed by the enzyme colorimetric method from Saike Biotechnology Co., Ltd. (Ningbo, China) using Olympus AU5821 from Beckman Coulter.

### Statistical analysis

The obtained data were analyzed by SPSS 26.0 statistical software(SPSS Inc., Chicago, IL USA), and P < 0.05 was considered to be significantly different. The categorical variables were expressed as number and percentage (n, %). The continuous variables were expressed as median and interquartile range (IQR, 25–75), D’Agostino-Pearson test was used to assess normality of data distribution. Use univariate logistic regression analysis to select the independent indicators, and the Akaike information criterion (AIC) of the multivariable logistic regression models was used to choose statistically significant variables. Expressed as estimated odds ratios (OR) and 95% confidence intervals (CI). The receiver operating characteristic (ROC) curve and the corresponding AUCs were used to evaluate the value of biomarkers to diagnose TPE above age 60.We also calculated sensitivity, specificity, positive predictive value (PPV), negative predictive value (NPV), positive predictive value (PLR), and negative predictive value (NLR) to measure the diagnostic accuracy.

## Results

### Participants

The study conducted at Ningbo First Hospital involved a total of 637 patients. After excluding 118 individuals based on specific criteria such as age, pregnancy, incomplete data, and unknown effusion etiology, a final cohort of 519 patients was analyzed, with 249 diagnosed as TPE and 270 as non-TPE. Details of the non-TPE group, including age distribution, are presented in **Table 1**. Among the non-TPE patients, 268 were between 18 and 59 years old, while 251 were 60 years old and above, as illustrated in **Figure 1**. The demographic, clinical, and laboratory characteristics of the entire study population are succinctly outlined in **Table 2**.

### The results of univariate and multivariate logistic regression analysis for distinguishing TPE from non-TPE according to age

The cutoff values of the variables were determined using Youden’s indices. **Supplementary File 1**: **Supplementary Tables S1**, all variables were analyzed using the Mann-Whitney U test between TPE and non-TPE groups. Furthermore, the results of the univariate logistic analysis, which included 7 variables and their ratios, were presented in **Supplementary File 1**: **Supplementary Tables S1**, **2**. To explore the diagnostic value of biomarkers, 7 variables with an AUC>0.80 were selected for multiple regression analysis. The AIC method was used to stepwise select the regression model, identifying the 3 most valuable variables for distinguishing TPE from non-TPE based on age (**Table 3**).

**Table 3:** Multivariate logistic regression analysis of the clinical characteristics for discriminating TPE from non-TPE according to age.

Variables	cut-off	Multivariate analysis OR (95%C1)	P value
Effusion ADA			
≤59	24.95(U/L)	0.113(0.05-0.253)	<0.001
≥60	20.65(U/L)	0.049(0.017-0.146)	<0.001
IGRA			
≤59	26.5(pg/ml)	0.089(0.040-0.195)	<0.001
≥60	24(pg/ml)	0.035(0.012-0.105)	<0.001
Effusion LDH/Effusion ADA			
≤59	20.16	0.170(0.077-0.375)	<0.001
≥60	17.86	0.195(0.071-0.533)	0.001

### Correlations between age and pleural fluid biomarker levels in the entire group and in patients with TPE only

The correlation of Effusion ADA and LDH were not significant with age, and the analysis revealed weak but significant negative correlations between the levels of IGRA or Effusion LDH/Effusion ADA and patients’ age in the entire group. However, in a subanalysis which included only patients with TPE, the correlations between age and biomarker levels were not significant (**Table 4**).

**Table 4:** Correlations between age and pleural fluid biomarker levels in the entire group and in patients with TPE only.

	Variables	Entire group (n=519)		TPE group (n=249)	
		R Spearman	P value	R Spearman	P value
Age and	Effusion ADA	-0.061	0.162	0.068	0.288
	IGRA	-0.224	< 0.001	-0.085	0.183
	Effusion LDH	0.085	0.054	0.089	0.160
	EffusionLDH/Effusion ADA	0.155	< 0.001	0.002	0.977

### The diagnostic performance of indicators for TPE according to age

**Figure 2:**
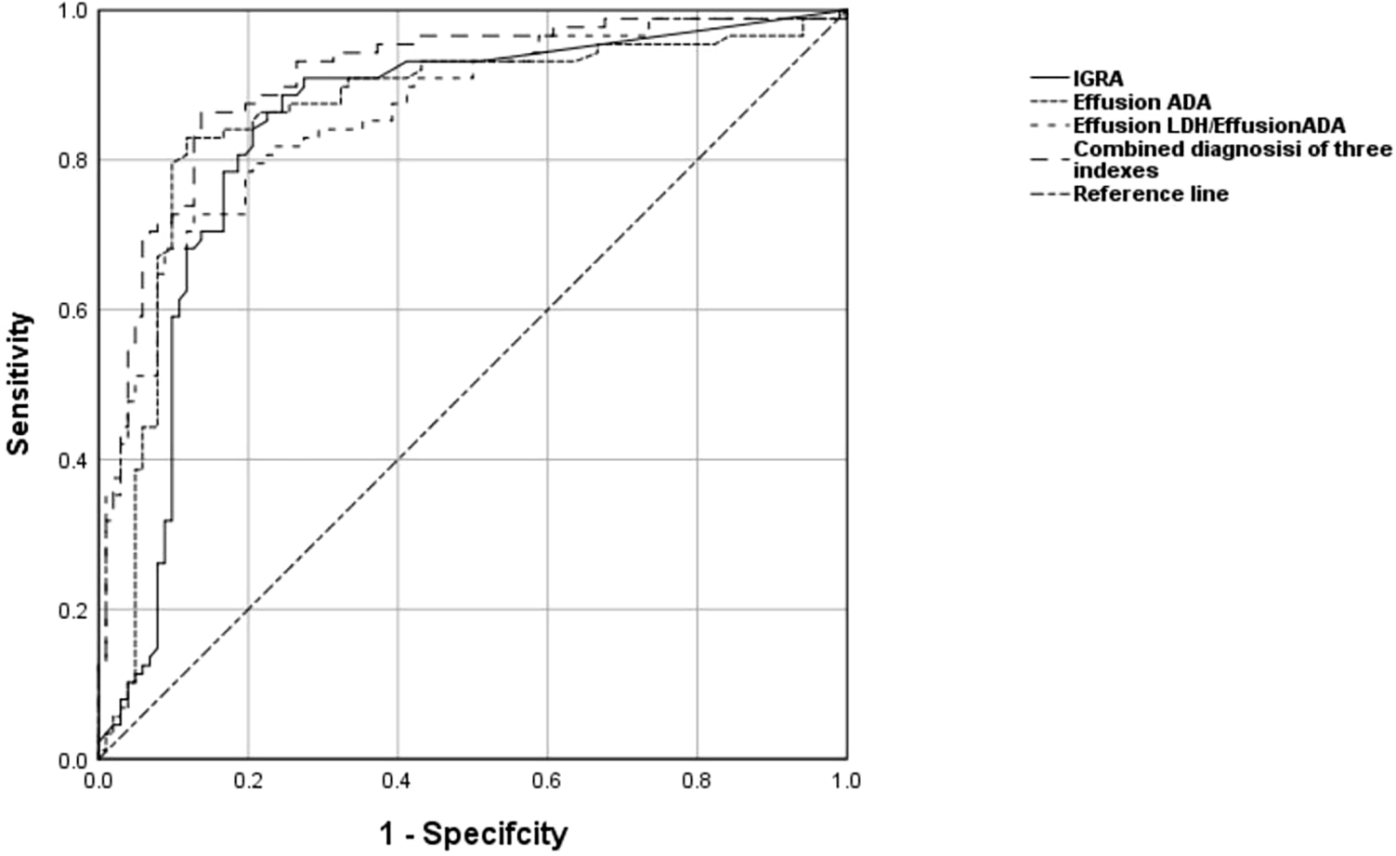
The levels of Effusion ADA, IGRA, Effusion LDH/Effusion ADA, and the combined diagnosis of the three indexes are used to discriminate TPE from non-TPE in the aged 60 and above. ROC curve of Effusion ADA, IGRA, Effusion LDH/Effusion ADA and the combined diagnosis of the three indexes prediction probability of distinguishing TPE from non-TPE.

To distinguish TPE from non-TPE in individuals aged 60 years and older, the diagnostic performance of all indicators was assessed using Receiver Operating Characteristic (ROC) analysis. An AUC greater than 0.80 was considered a valid marker. The detailed comparative diagnostic reference indicators and their corresponding performance were listed in **Table 5**. The AUCs of effective indexes for differentiating TPE from non-TPE based on age were as follows: Effusion ADA (0.846, 95% CI (0.791-0.901)(≤59), (0.859, 95% CI 0.800-0.918)(≥60); IGRA (0.850, 95% CI 0.799-0.901)(≤59), (0.839, 95% CI (0.778-0.901)(≥60); Effusion LDH/effusion ADA (0.849, 95% CI 0.800-0.899)(≤59), (0.861, 95% CI (0.808-0.914))(≥60) and [0.900 (0.858-0.934)(≤59), 0.925(0.885-0.954)(≥60)] for combined diagnosis of the three indexes (**Table 5**; **Figure 2**). Compared to Effusion ADA, the IGRA and Effusion LDH/Effusion ADA demonstrated a good diagnostic accuracy for TPE(≥60) in terms of sensitivity (88.64%, 82.95, 72.73), and specificity (75.49%, 88.24, 87.25), respectively. However, the combined diagnosis of three indexes yielded the highest diagnostic accuracy for TPE(≥60) with sensitivity of 85.23%, and specificity of 89.57% (**Table 5**; **Figure 2**).

**Table 5:** Diagnostic performance of the indexes based on ROC in differentiating TPE from non-TPE according to age.

Indexes	Ages	AUC(95%CI)	Sensitivity(%)(95%CI)	Specificity(%)(95%CI)	PPV(%)(95%CI)	NPV(%)(95%CI)	LR+(%)(95%CI)	LR-(%)(95%CI)
Effusion ADA	Aged 18-59	0.846(0.791-0.901)	77.64(70.4-83.8)	85.05(76.9-91.2)	88.7(83.2-92.5)	71.7(65.2-77.3)	5.19(3.3-8.2)	0.26(0.2-0.4)
	Aged 60-98	0.859(0.800-0.918)	82.95(73.4-90.1)	88.24(80.4-93.8)	85.9(78.0-91.3)	85.7(79.0-90.5)	7.05(4.1-12.1)	0.19(0.1-0.3)
IGRA	Aged 18-59	0.850(0.799-0.901)	83.23(76.5-88.6)	82.24(73.7-89.0)	87.6(82.3-91.4)	76.5(69.6-82.3)	4.69(3.1-7.1)	0.2(0.1-0.3)
	Aged 60-98	0.839(0.778-0.901)	88.64(80.1-94.4)	75.49(66.0-83.5)	75.7(68.8-81.6)	88.5(81.0-93.3)	3.62(2.6-5.1)	0.15(0.08-0.3)
Effusion LDH/ Effusion ADA	Aged 18-59	0.849(0.800-0.899)	78.88(71.8-84.9)	82.24(73.7-89.0)	87(81.5-91.0)	72.1(65.5-77.9)	4.44(2.9-6.7)	0.26(0.2-0.4)
	Aged 60-98	0.861(0.808-0.914)	72.73(62.2-81.7)	87.25(79.2-93.0)	83.1(74.5-89.3)	78.8(72.3-84.0)	5.71(3.4-9.6)	0.31(0.2-0.4)
Combined diagnosis of three indexes	Aged 18-59	0.900(0.858-0.934)	86.25(79.9-91.2)	85.98(77.9-91.9)	90.2(85.1-93.7)	80.7(73.8-86.1)	6.15(3.8-9.9)	0.16(0.1-0.2)
	Aged 60-98	0.925(0.885-0.954)	85.23(76.1-91.9)	89.57(83.8-93.8)	81.5(73.6-87.5)	91.8(87.1-94.9)	8.17(5.2-12.9)	0.16(0.10-0.3)

## Discussion

As the elderly population aged 60 years and above to grow, research focusing on diagnostic methods for tuberculous pleurisy in this age group becomes increasingly crucial. However, elderly patients with TPE often have weakened immune function, are frequently complicated by other underlying diseases, and present with atypical clinical manifestations and complex imaging features. This results in a high incidence (Wang and Wang, 2021; C. Antimicrobial Resistance, 2022), prevalence (C. Antimicrobial Resistance, 2022) and mortality rate (C. Tuberculosis Surveillance, 2016; Guthmann et al., 2020), along with a low etiology positive rate (Li et al., 2021), leading to easy delays in diagnosis and treatment (Guthmann et al., 2020). Therefore, further diagnostic investigations targeting elderly patients (≥60 years old) with tuberculous pleurisy are of paramount importance.

ADA and IGRA were critical marker for diagnosing tuberculous pleurisy. Previous studies have highlighted that the diagnostic value of ADA may be impacted as age increases (Lee et al., 2014; Porcel, 2018; Jiang et al., 2020), whereas IGRA may remain unaffected (Li et al., 2023; Zheng et al., 2023). Our results revealed weak or no significant differences in the levels of these biomarkers between younger and older patients. Additionally, a *post-hoc* analysis of two independent prospective blinded studies supported our findings (Jiang et al., 2020). However, subgroup analysis revealed a slight decrease in the cutoff values of Effusion ADA (20.65 vs 24.95 U/L), IGRA (24 vs 26.5 pg/ml), and Effusion LDH/Effusion ADA (17.86 vs 20.16) in the older age group (60-98 years) compared to the younger age group (17-59 years). This discrepancy may be partly related to various factors such as study inclusion criteria, sample size, and age distribution. Therefore, further investigation is needed to fully understand the impact of age-related changes in pleural biomarker levels and the underlying mechanisms.

In the subgroup of individuals aged 60 years and older, ADA effectively distinguished tuberculous pleural effusion (TPE) from non-TPE cases with an AUC of 0.859 (0.800-0.918), indicating strong clinical diagnostic utility, consistent with literature reports (Li et al., 2023). However, Our analysis of patients with exudative pleural effusion in the 60-98 age group revealed an optimal TPE cut-off value of 20.65 U/L, which was lower than values reported in some previous studies (Bielsa et al., 2013; Jiang et al., 2020), which may be related to the fact that the previous literature did not classify ADA by age or local tuberculosis prevalence (Skouras and Kalomenidis, 2016; Chang et al., 2018; Korczynski et al., 2019). Consequently, further research is needed to refine the optimal ADA threshold and discover more precise diagnostic markers for elderly patients.

Recent studies have demonstrated significant differences in IGRA results between TPE and non-TPE groups, including cases of malignant pleural effusion, pneumonia, cirrhosis, and so on (Fei et al., 2023). Analysis of the data from this study revealed that IGRA presents a strong diagnostic advantage in individuals aged 60-98, showing high sensitivity (88.64%) and specificity (75.49%), results that closely align with Zhishu Li’s previous research (Mollo et al., 2017). The specificity of 75.49% observed in this study is slightly lower than that reported in some other studies, possibly due to factors such as high comorbidity, sample size, and variations in age distributions (Jiang et al., 2020). However, immune function tests, including lymphocytes and their subsets in both peripheral blood and pleural effusion, were not conducted to assess peripheral and local lymphocyte function in the pleural cavity for a more comprehensive analysis of the influencing factors of IGRA and ADA (Keng et al., 2013). Therefore, when diagnosing tuberculous pleural effusion in this population, it is advisable to combine IGRA with other more specific biomarkers for a more accurate diagnosis.

The effusion LDH/ADA ratio was also evaluated to differentiate TPE from non-TPE (Beukes et al., 2021; Fei et al., 2023). Blakiston et al. established a cutoff value of 15.0 for this ratio, showing high sensitivity and specificity. Another study found that the effusion ADA/LDH ratio had a sensitivity of 81.13% and specificity of 83.67% at a cutoff of 14.29, with an AUC of 0.888 for distinguishing TPE from PPE (Gao et al., 2023). In our study, we determined a cutoff value of 17.86 (sensitivity: 85.23%, specificity: 89.57%) for the effusion LDH/effusion ADA ratio in diagnosing TPE in individuals aged 60 and above. The cutoff value is slightly higher compared to previous studies. Since ADA in pleural fluid mainly comes from macrophages and lymphocytes, a decrease in pleural fluid ADA with age may indicate age-related dysfunction of these cells as proposed by Hsu et al (Hsu et al., 1993). Notably, this is the limited, to our knowledge, to investigate effusion LDH/effusion ADA for distinguishing TPE from non-TPE in individuals aged 60 and above. Therefore, further prospective studies are warranted to validate our findings in the age group of 60 years and older. Additionally, we analyzed the values of several variables, including IGRA/ADA, LDH/IGRA, effusion LDH/IGRA, effusion ADA/ADA, and LDH/Infusion ADA. While IGRA/ADA, effusion ADA/ADA, and LDH/effusion ADA demonstrate some diagnostic value in the elderly population aged over 60 years, their diagnostic utility did not improve with the redundant calculation of the complex ratio. Consequently, we opted not to pursue further analysis.

While individual markers like IGRA, Effusion ADA, and Effusion LDH/Effusion ADA have shown promise in diagnosing TPE in elderly patients, combining multiple markers could offer even greater benefits in clinical practice. Previous research has indicated that using a combination of two or more markers is more accurate for TPE diagnosis than relying on a single marker alone (Aggarwal et al., 2015; Fenhua et al., 2021). In our study, we found that a combination of IGRA, Effusion ADA, and Effusion LDH/Effusion ADA produced the highest AUC [0.925, 95% CI (0.889-0.961)], with a sensitivity of 85.23% and specificity of 89.57%, surpassing other indicators for diagnosing TPE in elderly patients. This combination also demonstrated a NPV of 91.80%, suggesting a low probability of misdiagnosing non-TPE cases. The integration of PLR and NLR allows for a comprehensive evaluation of sensitivity, specificity, PPV, and NPV in disease diagnosis, making them valuable and independent indexes for identifying elderly patients with TPE (≥ 60 years old).

Effusion ADA, IGRA, and the combination of Effusion LDH/Effusion ADA demonstrate good diagnostic and differential diagnostic value in the age group of 60 years and older with exudative pleural effusion, especially in elderly patients suspected of having tuberculosis. This is particularly beneficial for patients who are unwilling to undergo medical thoracoscopy or pleural biopsy, or for medical units without thoracoscopy equipment. Additionally, utilizing ADA, IGRA, and Effusion LDH/Effusion ADA testing can eliminate the discomfort and risks associated with invasive procedures, reduce the financial burden on patients, and enhance the accuracy of tuberculosis diagnosis.

The study’s limitations include its retrospective, single-center design, emphasizing the necessity for additional prospective, multicenter studies involving diverse populations for validation. Furthermore, the exclusion of potential biomarkers like IL-27, IL-32, TNF-α, and CXCL9 in the analysis could enhance diagnostic accuracy. Given the regional focus on Chinese patients and the varying TB incidence globally, future multicentric, prospective studies with comprehensive data are essential to corroborate these findings.

## Conclusion

Combined detection of IGRA, Effusion ADA, and Effusion LDH/Effusion ADA can improve the diagnostic efficacy of tuberculous pleurisy in individuals aged 60 and above.

## Data Availability

The original contributions presented in the study are included in the article/**Supplementary Material**. Further inquiries can be directed to the corresponding author.

## Supplementary material

Supplementary Table 1 Univariate logistic regression analysis of the clinical characteristics for discriminating TPE from PPE(age ≤ 59).

Supplementary Table 2 Univariate logistic regression analysis of the clinical characteristics for discriminating TPE from PPE(age≥60).
