# Supplementary material for: Diagnostic value of effusion adenosine deaminase, γ-interferon release assay and effusion lactatedehy drogenase/effusion adenosine deaminase for tuberculous pleural effusion in patients aged 60 years and above
Source: Front Cell Infect Microbiol. 2024 Oct 9;14:1444238. doi: 10.3389/fcimb.2024.1444238 (PMC11496274; doi:10.3389/fcimb.2024.1444238)
Supplement: Supplementary Table 1 — Univariate logistic regression analysis of the clinical characteristics for discriminating TPE from PPE(age ≤ 59). [file Table1.docx]

| Table S1 Univariate logistic regression analysis of the clinical characteristics for discriminating TPE from PPE(age≤59) | | | | |
| --- | --- | --- | --- | --- |
| Variables | Cut-off | AUC(95%CI) | Univariate analysis OR (95% CI)P value | *P* value (Mann- Whitney U test) |
| IGRA | 12.00 | 0.821 | 1.010(1.006-1.014)0.000 | 0.000 |
| crp | 92.26 | 0.49 | 0.994(0.988-0.999)0.019 | 0.826 |
| esr | 39.50 | 0.588 | 1.015(1.002-1.027)0.025 | 0.03 |
| TP | 74.80 | 0.571 | 1.030(1.001-1.060)0.045 | 0.083 |
| ALB | 30.60 | 0.498 | 1.002(0.948-1.059)0.945 | 0.906 |
| ADA | 10.35 | 0.645 | 1.095(1.020-1.177)0.013 | 0.000 |
| LDH | 169.50 | 0.523 | 0.998(0.995-1.001)0.134 | 0.541 |
| Effusion TP | 48.95 | 0.631 | 1.072(1.029-1.116)0.001 | 0.002 |
| Effusion ALB | 26.00 | 0.628 | 1.077(1.020-1.136)0.007 | 0.002 |
| Effusion ADA | 24.95 | 0.821 | 1.056(1.033-1.080)0.000 | 0.000 |
| Effusion LDH | 222.50 | 0.543 | 1.000(1.000-1.000)0.329 | 0.273 |
| Effusion GLU | 5.74 | 0.321 | 0.961(0.915-1.010)0.120 | 0.000 |
| IGRA/ADA | 1.61 | 0.803 | 1.059(1.029-1.090)0.000 | 0.000 |
| LDH/IGRA | 14.27 | 0.828 | 0.961(0.950-0.972)0.000 | 0.000 |
| Effusion LDH/IGRA | 20.28 | 0.808 | 0.991(0.987-0.994)0.000 | 0.000 |
| Effusion ADA/ADA | 2.00 | 0.755 | 0.812(1,111-1.651)0.000 | 0.000 |
| LDH/Effusion ADA | 8.78 | 0.837 | 1.911(0.762-0.865)0.000 | 0.000 |
| Effusion LDH/Effusion ADA | 20.15 | 0.816 | 0.952(0.934-0.970)0.000 | 0.000 |
| Abbreviations:TP Total protein,ALB albumin ADA adenosine deaminase, LDH lactatedehy drogenase, CRP C-reactive protein, ESR erythrocyte sedimentation rate,IGRA Interferon-γ release assay | | | | |

| Table S2 Univariate logistic regression analysis of the clinical characteristics for discriminating TPE from PPE(age≥60) | | | | |
| --- | --- | --- | --- | --- |
| Variables | Cut-off | AUC(95%CI) | Univariate analysis OR (95% CI)P value | *P* value (Mann- Whitney U test) |
| IGRA | 24.00 | 0.839 | 24.024(10.815-53.364)0.000 | 0.000 |
| crp | 12.12 | 0.547 | 2.067(1.081-3.955)0.028 | 0.264 |
| esr | 25.50 | 0.637 | 1.014(1.003-1.026)0.017 | 0.001 |
| TP | 67.90 | 0.560 | 1.033(1.003-1.064)0.032 | 0.154 |
| ALB | 35.95 | 0.446 | 0.954(0.900-1.011)0.112 | 0.196 |
| ADA | 11.05 | 0.687 | 1.177(1.081-1.281)0.000 | 0.000 |
| LDH | 162.50 | 0.529 | 0.998(0.995-1.001)0.312 | 0.489 |
| Effusion TP | 41.25 | 0.622 | 1.012(0.991-1.034)0.249 | 0.004 |
| Effusion ALB | 20.85 | 0.572 | 1.024(0.0985-1.066)0.232 | 0.089 |
| Effusion ADA | 20.65 | 0.859 | 1.088(1.059-1.117)0.000 | 0.000 |
| Effusion LDH | 224.50 | 0.578 | 1.000(0.999-1.000)0.411 | 0.065 |
| Effusion GLU | 7.89 | 0.548 | 1.080(0.971-1.200)0.155 | 0.255 |
| IGRA/ADA | 2.51 | 0.817 | 1.083(1.045-1.283)0.000 | 0.000 |
| LDH/IGRA | 6.15 | 0.841 | 0.962(0.949-0.976)0.000 | 0.000 |
| Effusion LDH/IGRA | 14.24 | 0.815 | 0.982(0.974-0.989)0.000 | 0.000 |
| Effusion ADA/ADA | 1.79 | 0.805 | 1.679(1.319-2.136)0.000 | 0.000 |
| LDH/Effusion ADA | 11.81 | 0.838 | 0.933(0.905-0.962)0.000 | 0.000 |
| Effusion LDH/Effusion ADA | 17.85 | 0.861 | 0.928(0.903-0.953)0.000 | 0.000 |
| Abbreviations:TP Total protein,ALB albumin ADA adenosine deaminase, LDH lactatedehy drogenase, CRP C-reactive protein, ESR erythrocyte sedimentation rate,IGRA Interferon-γ release assay | | | | |
|  | | | | |
